# Rethinking speech sound disorder (SSD) in non‐syndromic cleft lip and palate: The importance of recognizing phonological and language difficulties

**DOI:** 10.1111/1460-6984.13151

**Published:** 2025-01-17

**Authors:** Stephanie van Eeden, Cristina McKean, Helen Stringer

**Affiliations:** ^1^ School of Education Communication and Language Sciences Newcastle University Newcastle upon Tyne UK

**Keywords:** cleft palate, language, phonology, speech sound disorder

## Abstract

**Background:**

Children born with cleft palate ± lip (CP ± L) are at risk of speech sound disorder (SSD). Up to 40% continue to have SSD at age 5–6 years. These difficulties are typically described as articulatory in nature and often include cleft speech characteristics (CSC) hypothesized to result from structural differences. In non‐CP ± L SSD comorbidity with language difficulties is often reported. There is growing evidence of concomitant language difficulties in children with CP ± L and of a higher prevalence of developmental speech errors in children compared with non‐CP ± L peers. The impact of underlying phonological and language skills on speech production in children with CP ± L is poorly understood.

**Aims:**

To investigate language outcomes in children with CP ± L and the relationship to speech production, by answering the following research questions: (1) Does the profile of language skills in children with CP ± L differ from normative samples? (2) Do children with CP ± L and SSD have poorer language skills than those with typically developing speech? (3) Is there an association between language skills and speech profile in children with CP ± L at age 5–8 years?

**Methods & Procedures:**

In this prospective cross‐sectional, observational study, 95 participants were recruited from regional cleft lip and palate services in the UK. They were aged 5;0–7;11 with non‐syndromic CP ± L. Those with a syndromic diagnosis, global learning disability, sensorineural hearing loss and first language other than English were excluded. Assessments of speech (Diagnostic Evaluation of Articulation and Phonology—DEAP) and language (Clinical Evaluation of Language Fundamentals—5th UK edition—CELF) were completed. Language outcomes were analysed and compared with normative samples and according to speech error analysis.

**Outcomes & Results:**

Average language scores were within the expected range. For those presenting with SSD, language scores were significantly lower than those with typically developing speech. Analysis of speech errors showed four distinct speech profiles: typical speech, CSC only, developmental speech characteristics (DSC), and combined CSC + DSC. Language scores were lower for participants with DSC (±CSC). A significant association was found between the presence of CSC + DSC and expressive language outcomes (odds ratio (OR) = 10.82; 95% confidence interval (CI) = 2.42, 48.32, *p* = 0.002).

**Conclusions & Implications:**

An association between language skills and speech production was observed. The distribution of speech errors in children with CP ± L varied with a high level of DSC as well as CSC. Those with CSC + DSC had significantly lower language scores than those with typically developing speech or CSC only. Speech and language therapists working with this caseload should be alerted to potential ongoing phonological and language difficulties in children presenting with this profile.

**WHAT THIS PAPER ADDS:**

**What is already known on this subject**

- Children born with CP ± L are known to be at risk of SSD. There is also evidence of language delay in the early years. Evidence for persistent language difficulties is equivocal but some studies have shown a higher than average prevalence of developmental phonological errors in addition to cleft articulation errors in speech production.

**What this paper adds to the existing knowledge**

- This study investigates links between speech development and language skills in children aged 5–8 years with non‐syndromic CP ± L. It adds to our understanding of the nature of SSD in children with CP ± L and in particular the relationship between language skills and speech production. It shows that children with CP ± L have varied speech profiles and that those presenting with delayed phonological processes are also at risk of language difficulties.

**What are the potential or actual clinical implications of this work?**

- Speech and language therapists working with children with CP ± L should be aware of the varied nature of SSD in this population. They should be alert to the need for additional assessment of language for those presenting with delayed phonological processes and the implications this has for educational attainment.

## INTRODUCTION

There is a growing body of evidence indicating that children with speech and language difficulties experience lower educational attainment, poorer psychosocial development and restricted later life chances compared with children with typically developing speech and language (Clegg et al., 2005; Wren et al., 2023; Ziegenfusz et al., 2022). Children born with cleft palate ± lip (CP ± L), as a subgroup of children with speech and language difficulties, are reported to be at greater risk of speech difficulties (Britton et al., 2014), lower literacy skills (Lancaster et al., 2022), poor academic achievement and psychosocial difficulties (Hunt et al., 2005; Watkins et al., 2018) compared with non‐CP ± L peers. Achieving speech that is understandable and acceptable is the primary outcome measure following surgical repair of a cleft palate in infancy. The speech of children with CP ± L is typically reported in terms of velopharyngeal function and articulation of speech sounds (Chapman & Willadsen, 2011). The term *articulation* describes the execution of motor skills to produce speech sounds and, in addition to velopharyngeal function, is the main focus of observations of cleft speech both clinically and in research. When describing speech errors in people with CP ± L it is commonly understood that they are a result of the craniofacial structural anomaly which may have phonological consequences (Alighieri et al., 2020; Harding‐Bell & Howard, 2011). More recently there have been reports of high levels of the type of developmental speech sound errors seen in the broader population (Nachmani et al., 2022; Willadsen et al., 2019) and calls to re‐evaluate the nature of speech sound disorder (SSD) related to CP ± L (Pereira & Sell, 2023; Southby, 2024). In line with a UK‐wide move towards a consensus of definitions for subtypes of SSD (Stringer et al., 2023), this paper will use SSD as an overarching term. Distinctions will be made between an articulation disorder being a motor difficulty (physical production of sounds) and a phonological disorder being a linguistic difficulty (using sounds in words to signal meaning). In viewing these subtypes as distinct, the underlying mechanisms leading to changes in speech production are more easily considered. These mechanisms may also help our understanding of the lower educational attainment seen in the CP ± L population by allowing greater reflection of the impact of a potential linguistically based SSD in some children on language and later literacy skills which are key to academic achievement (Hayiou‐Thomas et al., 2017).

Efforts to understand the underlying mechanisms of SSD which persist beyond the age of 5 years in children with CP ± L have been minimal. Hypotheses relating to hearing have been inconclusive (Baker et al., 2021; Lohmander et al., 2021) as have those relating to speech processing (Southby et al., 2021). Other potential underlying skills which have been related to speech development in the wider population are language, auditory, and cognitive skills (Dawes & Bishop, 2009; Gooch et al., 2016; Kuhl et al., 2005). This paper will focus on the relationship of language skills to speech development.

Classification of SSD in CP ± L is often reported to have a known aetiology due to the diagnosis of cleft palate and therefore connected to an anatomical‐sensory explanation of the resultant SSD. Other linguistically driven models of SSD are often overlooked. This is despite reports of concomitant language difficulties in children with CP ± L, especially in the early years (Lancaster et al., 2020). These reported language difficulties are often attributed to hearing difficulties and delayed and restricted babble due to palate repair taking place after the crucial early babble development stage seen in infants without CP ± L which can lead to continued restricted consonant inventories in early childhood (Baylis et al., 2020; Chapman et al., 2001; Hardin‐Jones & Chapman, 2014). In the wider SSD literature, comorbidities with other communication skills are frequently reported. Rates of comorbidity between SSD and language disorder can vary but one of the largest population studies reported that children with SSD were six times more likely to have a language disorder than children without SSD (Beitchman et al., 1986). Variability in the rates of comorbidity may depend on the phenotypical presentation of the SSD. Broomfield and Dodd (2004) reported on 320 children with different SSD subtypes and found rates of comorbidity with language disorder differed depending on the presenting SSD. For example, 17.5% of those with articulation disorder alone had receptive language difficulties compared with 40% of those with an inconsistent phonological disorder.

Research examining rates of comorbidity or the relationship between speech and language skills in children with CP ± L is limited to a few studies, with mostly small sample sizes. Willadsen (2013) compared Danish‐speaking toddlers (age 18 months) with CP ± L to age‐matched non‐cleft peers on measures of expressive vocabulary and phonetic inventories. The study showed an association between the phonetic inventory and lexical selectivity of vocabulary items in both groups (*r*_s_ = 0.877, *p* < 0.001 (CP ± L group); *r*_s_ = 0.867, *p* < 0.001 (non‐CP ± L group)). Children with CP ± L had reduced phonetic inventories compared with peers and the words they used were significantly different in terms of word initial consonants regarding both manner and place of articulation. For example, children with CP ± L were more likely to use words which began with nasal and velar consonants and less likely to use words which began with plosives, fricatives, or alveolar consonants. There was also a significant correlation between the number of different consonants used and productive vocabulary observed in a 45‐min play session with a parent. Similarly, a study from the United States of toddlers with and without CP ± L aged 21 and 27 months showed a link between their phonetic inventory and vocabulary size. The CP ± L group had a mean average of 50–80 fewer words at each time point than their non‐CP ± L peers with a specific association observed between words selected and the reduced use of obstruent sounds (Hardin‐Jones & Chapman, 2014).

Results from studies of older children have been equivocal. Klintö et al. (2015) found that 65.5% of 5‐year‐olds with unilateral cleft lip and palate (UCLP) had a vocabulary score 1 SD (standard deviation) below the normative mean on a test of narrative, but that there was no correlation with this and speech production. Conversely Pamplona et al. (2000) found that in 29 children aged 3–8 years, none of those with ‘compensatory articulation disorder’ had typically developing language, compared with 70% in the group with typically developing speech. Some larger CP ± L cohort studies, whilst not investigating comorbidities, have found high rates of developmental speech errors in participants, suggesting a level of phonological disorder and therefore a linguistic deficit, as opposed to an articulatory‐motor deficit. The ScandCleft study (Willadsen et al., 2019) reported over 50% of children aged 5 years with UCLP as having developmental errors including voicing, stopping, and fronting of consonants. A large retrospective study of nearly 800 Hebrew‐speaking participants with CP ± L aged between 3 and 25 years old found 31.5% to have phonological errors including errors that we would see in English‐speaking populations, namely, stopping, fronting and cluster reduction (Nachmani et al., 2022). This study found that it was more common to have developmental errors if participants also presented with cleft related errors described as ‘compensatory articulations’ than if they did not.

To further our understanding of speech and language development in children with CP ± L we need to know more about underlying and interactive mechanisms. Understanding of child development has moved forward with the onset of epidemiological, longitudinal and neurobiological studies (McKean et al., 2018). This has increased understanding of the role of innate biological constraints, how children adapt and how developmental pathways can change over time. One influential model which has helped shape our understanding comes from neuroconstructivist theory. Neuroconstructivist theorists see neonatal neural structures as undifferentiated, remaining flexible to process incoming information in the most efficient way available given innate genetic factors and influential environmental factors. The experience of development itself leads thereafter to more specific modular brain functions (Karmiloff‐Smith, 1998). Important to understanding this theory is the role of *interaction*, *compensation* and *timing*. Development in children with CP ± L is complex, with multiple interacting factors. Development is compromised at birth due to the anatomical difference of both the orofacial structures and auditory structures. The interaction of these and the timing of interventions and experiences across development may lead to a typical developmental trajectory or to compensations which result in later weaknesses in a number of aspects of development, including speech and language. Thomas and Karmiloff‐Smith (2005) argue that compensation is likely to have taken place if innate constraints on biological and neurological systems have impacted typical developmental pathways. In some children, this may result in surface‐level language appearing to have developed well, but with an underlying system that is impaired, constrained by compensation. This may lead to unstable foundations for speech and language development. The nature of a later, overt impairment emerges from the interaction of compensating systems with these impaired foundations. An example of this might be a child with literacy difficulties who on assessment shows intact oral language skills but deficits in phonological awareness, resulting from the interaction of family history, environment and/or an earlier sensory impairment (Mareschal et al., 2007).

With this theory in mind, along with the evidence of language deficits in some children with CP ± L, speech and language therapists (SLTs) working in this field must ensure that full assessment of all speech and language skills is carried out. We posit that an understanding of the potential interactions between speech and language over development will support more accurate differential diagnosis and more theoretically motivated and hence effective intervention strategies for both speech and language (Frizelle & McKean, 2022). Furthermore, undiagnosed language difficulties increase risks of social and educational difficulties (Clegg et al., 2005; McGregor, 2020; Ziegenfusz et al., 2022) which are often reported in the CP ± L literature, but are poorly understood in this population (Feragen et al., 2015; Fitzsimons et al., 2021; Gallagher & Collett, 2019). Detailed observations of different aspects of language development are needed to understand the prevalence of these difficulties and the impact on other areas of development such as speech production and academic achievement. At the very least these should include an understanding of the relationship between expressive and/or receptive language and other skills. The Cleft Language and Auditory Skills (CLAS) study, presented here, was developed to address this gap in the research (https://www.fundingawards.nihr.ac.uk/award/ICA‐CDRF‐2017‐03‐002). The CLAS study aimed to describe language and auditory skills in children with CP ± L aged 5–8 years and explore the relationship of these skills to speech development. Findings from this study will facilitate a greater understanding of factors associated with and underlying SSD in children with CP ± L and highlight pertinent data to collect in future longitudinal studies. This paper presents CLAS study findings regarding language skills and their relation to speech. Findings regarding auditory skills will be reported in a separate paper.

## AIM AND RESEARCH QUESTIONS

To investigate the relationship between speech development and language skills in children with CP ± L and answer the following research questions:

- Does the profile of language skills in children with CP ± L differ from normative samples?
- Do children with CP ± L and any subtype of SSD have poorer language skills than those with typically developing speech?
- Is there an association between language skills and speech profile in children with CP ± L at age 5–8 years?

## METHODS

### Ethics

Ethical approval for this study was gained through the NHS Research Ethics Committee (19/ES/0071) and the Health Research Authority in the UK.

### Design

This was a prospective, cross‐sectional observational study.

### Participants

Participants meeting the inclusion/exclusion criteria were recruited from regional cleft lip and palate services across the north of England. Families were given information about the study in clinic or sent information in the post with a follow‐up phone call from members of the Research and Development departments in each recruiting centre. Informed written consent to take part was taken from parents and from children where possible before contacting families to arrange an assessment date; 98 participants were recruited. Participants were included if they were aged 5 years 0 months to 7 years 11 months. This age range was chosen as it was representative of the early school years and ensured any observed SSD was characterized as persistent. Participants with a cleft of the palate only (CPO), UCLP or bilateral cleft lip and palate (BCLP) and if English was the main language spoken at home were included. Children were excluded if they had an additional syndromic diagnosis, a known learning disability or sensorineural hearing loss.

### Procedure

A battery of speech, language and auditory assessments was administered to all participants to fulfil the aims of the CLAS study (see Appendix A for a full list). This paper focuses on the results of the language assessments. Participants were seen at home or at school depending on family preference. Assessment sessions lasted between 1.5 and 2 hours including breaks. Breaks in assessment were designed to ensure participants were able to attend to the tasks to their best ability. They followed a school timetable pattern which included a longer break to play outside and have a drink and snack halfway through the session. If at home, the longer break was given at the same time and participants chose to either have something to eat and drink or to play away from the assessment table. All assessments were carried out by the lead author who is a qualified SLT.

### Measures

Speech was assessed using the Diagnostic Evaluation of Articulation and Phonology (DEAP) (see Appendix A). This assessment is a 50‐item single word naming test. The word list contains all 24 consonants in syllable‐initial and syllable‐final positions as well as all vowels and diphthongs in English. It has over 60 examples of high‐pressure consonants, which are vulnerable in speech development of children with CP ± L, across syllable‐initial and syllable‐final positions. It allows for five opportunities of examples of typical developmental errors. This coupled with the use of narrow phonetic transcription (Sell, 2005) allowed the researchers in this study to identify delayed phonological processes and cleft‐type speech processes alike, more so than assessments specific to the assessment of speech in the CP ± L population, such as the Great Ormond Street Assessment of Speech (Sell et al., 1999). Using this method, speech errors were classified as cleft speech characteristics (CSC) or developmental speech characteristics (DSC). CSC errors can be active or passive. Active CSC are compensatory articulatory errors. They can be further characterized as anterior CSC (e.g., dental, lateral, palatal productions of alveolar targets); posterior CSC (e.g., backing to velar or uvular); or non‐oral CSC (e.g., active nasal fricatives, glottal and pharyngeal production). Passive CSC are obligatory errors which are the result of a continued structural anomaly. This includes weak and nasalized consonants, nasal realization of plosives or fricatives and gliding of fricatives and affricates. For analysis of speech errors in this study, where a passive CSC resulted in a loss of phonemic contrast (i.e., frank nasal production of plosives or fricatives and gliding) these were counted as a CSC and analysed accordingly. DSC errors included phonological processes such as fronting velar sounds to alveolar, typically [t] for /k/; stopping fricatives (e.g., /s/ → [d]; /f/ → [b]); final consonant deletion (e.g., cat → [kæ]); cluster reduction (e.g., snow → [nəʊ]); difficulties with approximants (e.g., muddling /ɹ, j, w, l/).

The DEAP percentage consonants correct (PCC) score was used to determine a cut‐off for typically developing speech in this age group. PCC is primarily used as an indicator of severity of SSD (Shriberg & Kwiatkowski, 1982), but it has been used in the field of cleft lip and palate as a means of creating a binary cut‐off for those above and below 2 SD from the normative mean at a given age (Klintö et al., 2022). Therefore, in this study, children with a PCC of 85 or less were judged to not have speech typical for their age. For all children in the study this represented a score at the 5th centile or below and a standard score of 5 or below.

Analysis of speech showed that there were four distinct groups:

- Speech typical for age (TS group): Participants in this group had a PCC score of 86% or above with no articulation or delayed phonological errors as determined by the DEAP. For example, age‐appropriate developmental processes, such as using [f] for /θ/ or [v] for /ð/ or a labiodental approximant ([ʋ]) for /ɹ/.
- CSC group: Participants in this group had CSC errors which were regarded as significant. This was determined using the guidelines laid out in the Cleft Audit Protocol for Speech‐Augmented (CAPS‐A) (John et al., 2006). This is a commonly used tool in the UK for audit and research purposes and uses a traffic light system to categorize severity. For example, a child would be classed as a green or light green rating if they had no CSC or only dentalization or mild lateralization or palatalization of one or two sounds. An amber rating is given if there is a lateral or palatal production of more than two sounds, or if posterior or non‐oral productions are detected on only one or two sounds. The most severe rating of red is given for speech characterized by more than two sounds being produced as posterior or non‐oral. If a participant was rated as amber or red, they were classed as having a significant level of CSC. This was classified as an articulatory subtype of SSD.
- DSC group: The DEAP classifies all phonological processes by age at which they should have resolved. For this study, if a child continued to present beyond these ages with a DSC, they were allocated to this group regardless of the PCC score. This was classified as a phonological subtype of SSD.
- CSC + DSC group: Some participants presented with both CSC and DSC as described above and were therefore classed as having a combined articulation and phonological SSD.

[Note: *Backing to velar (e.g., /t/ to [k]/*. This process may be regarded as phonological in nature. However, it is a well‐documented CSC. For the purpose of this study, backing to velar was counted as a CSC, as cleft speech was the primary focus of the study, and this is in line with other studies (Willadsen et al., 2019).]

Inter‐ and intra‐rater reliability of the transcription was carried out. A total of 10 consecutive speech recordings were analysed by the lead author and one other SLT, both of whom had specialized in cleft lip and palate for over 10 years. These same recordings were analysed a further time by the lead author for intra‐rater reliability, 6 months after the final participant was seen. This represented a sample of 1404 consonants. A percentage agreement score, and two‐way mixed model intraclass correlation coefficient (ICC) were calculated for both inter‐ and intra‐rater reliability. The percentage of absolute inter‐rater agreement across 1404 consonants was 84%. The percentage of intra‐rater agreement was 88%. Disagreements were minor and mostly noted on subtle articulatory differences at the anterior level, for example, a pure alveolar /s/ compared with a slightly retracted /s/. A two‐way mixed model for ICCs showed good agreement for both measures (inter‐rater ICC = 0.773; intra‐rater ICC = 0.827).

Language skills were assessed using the Clinical Evaluation of Language Fundamentals—5th UK edition (CELF‐5^UK^) (see Appendix A). This assessment allowed the formulation of detailed language profiles to include receptive skills, expressive skills, and a breakdown of specific skills as determined by the following subtests: Sentence Comprehension (understanding spoken sentences of increasing complexity), Word Structure (use of correct morphology), Word Classes (understanding semantic relationships between words), Following Directions (following directions of increasing length and complexity of concepts), Formulated Sentences (producing grammatically and semantically correct sentences to describe a picture with a given target word), Recalling Sentences (repeating sentences of increasing length verbatim).

Information related to development in children with CP ± L and speech and language development in general was also collected to be included as potential confounders in data analysis. This comprised: cleft type, structurally related speech difficulties/history of speech surgery (Butterworth et al., 2023), gender (Norbury et al., 2016), socio‐economic status (Reilly et al., 2007), family history of speech and language difficulties (Eadie et al., 2015), non‐verbal skills (Wetherell et al., 2007) and history of hearing difficulties (Schönweiler et al., 1998). Details of how confounding variables were measured are given in Table 1.

**TABLE 1:** Participant details.

		*N* (%)	Missing data (*n*)	Mean (SD, minimum–maximum)	Median (IQR)	Mode
Male gender^a^		51 (54)	0	–	–	–
Cleft type^a^	UCLP	31 (32.5)	0	–	–	–
	BCLP	11 (11.5)		–	–	–
	CPO	53 (56)		–	–	–
Involvement of hard palate^a^	None	9 (10)	6	–	–	–
	Incomplete	32 (36)		–	–	–
	Complete	48 (54)		–	–	–
Age at time of primary palate repair^a^	6–12 months	79 (83)	0	–	–	–
	13–18 months	14 (15)		–	–	–
	> 18 months	2 (2)		–	–	–
History of hearing problems^a^		57 (61)	2	–	–	–
Family history of S&L difficulties^a^		24 (26)	3	–	–	–
Structurally related speech difficulties^b^/history of speech surgery^a^		15 (16)	0	–	–	–
Age at time of assessment (months)^a^		–	0	76.7 (9.0, 60–95)	75 (70–83)	72
Non‐verbal skills (quartile)^c^		–	0	–	3 (2–4)	4
Socio‐economic status (decile)^d^		–	0	–	4 (1–8)	1

*Note*: ^a^Participant details form completed by site principal investigator.

^b^ From the connected speech sample obtained during expressive language assessment subtests of the CELF‐5^UK^ (Wiig et al., 2013).

^c^ Raven's Coloured Progressive Matrices assessment (Raven, 1988).

^d^ Index of Multiple Deprivation decile for England derived from postcode (Ministry of Housing, Communities and Local Government, 2019).

### Data analysis

Data were analysed using IBM SPSS v28. Descriptive statistics including mean scores, SD and range were compiled for composite receptive and expressive language scores. This was also done for all subtests and compared with data from the normative samples published in the CELF‐5^UK^ (Wiig et al., 2013). A percentage of participants with language scores falling 1 SD below the mean (i.e., scores < 85) was calculated. This represents scores below average which the CELF‐5^UK^ Examiners Manual suggests is an indicator that a child is likely to be struggling at school through potential on‐going language difficulties (p. 121).

A one‐way analysis of variance (ANOVA) was used to analyse differences between participants with SSD and those with typically developing speech. Linear regression explored the association between receptive and expressive language skills and speech production measured by PCC. The model was adjusted for the following co‐variables: cleft type, structurally related speech difficulties/history of speech surgery, language skills at 3 years, and family history. Age at assessment was also included as the PCC measure is not standardized.

ANOVA analyses were carried out with Bonferroni corrections to investigate the relationship between the four speech profiles and language. Following these initial analyses, post‐hoc analyses investigated the association between different SSD subtypes and language outcomes further, employing logistic regression to explore the risk of low language scores (1 SD below the mean) for each SSD subtype compared with those with speech typical for their age.

## RESULTS

### Participants

A total of 98 participants were recruited to the study. Three families did not respond to the invitation to attend the assessment day; therefore, data were gathered on 95 participants in total. When compared with data from the national UK audit database and other studies, this sample was representative in terms of cleft type, gender (The Cleft Registry and Audit Network (CRANE) Annual Report, 2023) and history of hearing difficulties (Baker et al., 2021). Most of the participants were from lower socio‐economic backgrounds, indicated by multiple deprivation indices obtained from postcodes. Almost 40% had received some therapy for cleft articulation problems as indicated by their local SLTs before the time of assessment (see Britton et al., 2014, for a comparison). The majority (83%) had their primary palate repair before the age of 12 months as is standard protocol in the UK. Five participants had ongoing velopharyngeal incompetence (VPI). Of these, three had already undergone secondary surgery to correct this. In total, 17 participants either had VPI or had a history of VPI post primary palate repair. Details of the participant characteristics are shown in Table 1.

### Language profiles compared with the normative sample

Average scores for the whole group for both receptive and expressive language composite scores were only slightly below the normative mean of 100 on the CELF‐5^UK^. The mean receptive language score was 97.9 (SD = 14.6, minimum–maximum = 67–134). The mean expressive language score was 96.4 (SD = 14.4, minimum–maximum = 57–128). Scores 1 SD below the mean (i.e., scores < 85) were analysed to determine the level of potential risk of ongoing language difficulties compared with the normative population; 25% of the group fell below this score for receptive language and 24% for expressive language. This is 8–9% more than would be expected.

Scores across all participants for four out of six subtests from the CELF‐5^UK^ were lower than the normative mean scaled score of 10. Details are shown in Table 2. Data showed that for all areas of language assessed (except for Word Structure) more participants with CP ± L had scores at least 1 SD below the mean than expected in the normative population, where only 16% are likely to fall below this level. For the Formulated Sentences subtest, which was observed to be the most difficult task for this group, 40% fell below 1 SD, 24% more than would be expected.

**TABLE 2:** Descriptive statistics for scaled scores from language subtests (from CELF‐5^UK^ (Wiig et al., 2013) for all participants.

	*n*	Mean (SD, minimum–maximum)	Score < 1 SD (*n*, %)
Sentence comprehension	95	10.0 (3.2, 1–17)	18 (19%)
Word structure	95	10.9 (3.2, 1–18)	12 (13%)
Word classes	95	9.6 (2.8, 4–17)	25 (26%)
Following directions	95	9.3 (2.8, 4–17)	24 (25%)
Formulated sentences	95	7.9 (2.9, 1–15)	38 (40%)
Recalling sentences	95	9.0 (2.4, 4–16)	26 (27%)

### Language profiles of those with and without SSD

The mean average PCC from the DEAP assessment for the whole group was 86% (SD = 14.7, minimum–maximum = 31–100); the median was 90% (IQR = 79–97). A total of 40 participants (42%) had a PCC of 85% or below and therefore were classed as having a SSD.

Receptive and expressive language scores were lower for participants with SSD. ANOVA analyses showed that there were statistically significant group differences between those with and without SSD for expressive language skills, including the subtests of Word Structure and Formulated Sentences (Table 3).

**TABLE 3:** Descriptive statistics and analysis of variance (ANOVA) analysis comparing scores, language indices and subtests (from CELF‐5^UK^; Wiig et al. 2013) between participants with and without SSD.

	No SSD (≥ 86%) (*n* = 55)	SSD (≤ 85%) (*n* = 40)	ANOVA		
	Mean (SD, minimum–maximum)	Mean (SD, minimum–maximum)	*F*(1, 93)	*p*	*η* ^2^ ^**^
Receptive language index	100.3 (14.7, 74–134)	94.6 (13.9, 67–123)	3.64	0.059	0.038
Expressive language index	99.2 (12.9, 62–128)	92.4 (15.5, 57–126)	5.46	0.022^*^	0.055
Sentence comprehension	10.5 (2.9, 5–16)	9.2 (3.6, 1–17)	3.87	0.052	0.040
Word Structure	11.6 (2.7, 3–18)	10.1 (3.7, 1–17)	5.28	0.024^*^	0.054
Word Classes	10.0 (2.9, 5–17)	9.0 (2.5, 4–16)	3.28	0.073	0.034
Following directions	9.4 (2.9, 4–17)	9.1 (2.7, 4–14)	0.34	0.564	0.004
Formulated sentences	8.5 (2.9, 2–15)	7.1 (2.8, 1–14)	6.14	0.015^*^	0.062
Recalling sentences	9.4 (2.2, 5–16)	8.6 (2.5, 4–15)	2.76	0.100	0.029

*Note*: ^*^Statistically significant (*p* < 0.05); ^**^effect size: small effect = 0.01; medium effect = 0.06; large effect = 0.14.

Regression analyses were used to investigate associations between speech and language skills. In a simple linear regression, expressive language scores significantly predicted speech outcomes, as measured by percent consonants correct (PCC) explaining a small amount of variance (*R* ^2^ = 0.05, B = 0.23, *F*(1, 94) = 4.89, *p* = 0.03). That is, for each increase of one standard score in expressive language, the child's PCC increased by 0.23. There was less evidence for receptive language as a predictor of speech outcome (*R* ^2^ = 0.04, *B* = 0.020, *F*(1, 94) = 3.62, *p* = 0.06). However, after adjusting for cleft type, structurally related speech difficulties/history of speech surgery, family history of speech and language difficulties, history of conductive hearing loss and age at assessment, neither receptive nor expressive language scores predicted PCC (Table 4).

**TABLE 4:** Linear regression analysis exploring predictors of speech (percent consonant correct (PCC) from DEAP assessment; Dodd et al., 2002).

	*B*	*t*	95% CI	*p*‐value
(Constant)	55.97	4.08	28.71, 83.23	< 0.001
Receptive language	0.01	0.04	−0.27, 0.28	0.970
Expressive language	0.12	0.84	−0.16, 0.39	0.403
Cleft type	−4.42	−3.54	−6.90, −1.94	< 0.001^*^
Structurally related speech difficulties/history of speech surgery	−11.58	−3.33	−18.49, −4.66	0.001^*^
Family history of speech and language difficulties	−5.44	−1.74	−11.69, 0.80	0.087
History of conductive hearing loss	−1.21	−0.43	−6.78, 4.36	0.668
Age at assessment	0.38	2.52	0.08, 0.69	0.014^*^

*Note*: *R* ^2^ = 0.35.

^*^Statistically significant (*p* < 0.05).

### Language skills in relation to speech profiles

A total of 39% of participants had developed speech sounds typical for their age; 35% presented with cleft articulation errors or CSC; 43% presented with phonological errors or DSC. There was some overlap between these last two groups resulting in the four distinct speech profiles discussed in the methods section:

- Speech typical for age (TS group).
- CSC only.
- DSC only.
- CSC + DSC.

**FIGURE 1:**
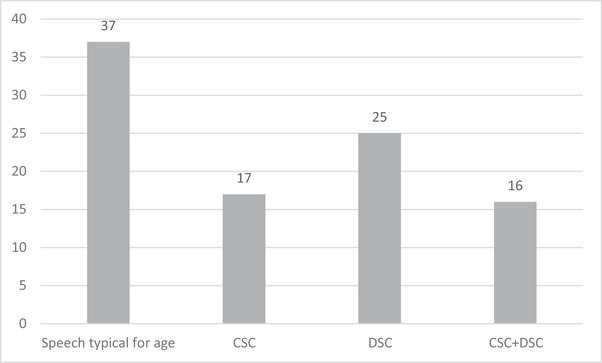
Number of participants with speech typical for age, cleft speech characteristics (CSC) only, developmental speech characteristics (DSC) only, and CSC + DSC.

The distribution of these profiles is shown in Figure 1.

A further breakdown of speech errors is shown in Table 5. This shows that those participants with typical speech for their age (*n* = 37) included 12 (32.4% of this group) with anterior errors such as dentalization; those with DSC only (*n* = 25) included 11 (44% of this group) with mild anterior errors not classed as a significant CSC using the CAPS‐A criteria (John et al., 2006); those with CSC only (*n* = 17) included 8 (47%) with significant anterior errors, 4 (23.5%) with posterior errors and 5 (29.4%) with non‐oral errors. Of note, 75% (*n* = 12) of the group who presented with both CSC and DSC had a predominant non‐oral CSC pattern. A highly significant and large effect of group was seen across the spread of different error patterns (*χ* ^2^ (9, *n* = 95) = 79.312, *p* ≤ 0.001, *Φ* = 0.914).

**TABLE 5:** Distribution of the predominant pattern of cleft speech characteristics (CSC) in individual participants with speech typical for age, CSC only, developmental speech characteristics (DSC) only, and CSC + DSC.

		Typical speech for age	CSC only	DSC only	CSC + DSC	Total
Predominant pattern of CSC	None	25	0	14	0	39
	Anterior	12	8	11	2	33
	Posterior	0	4	0	2	6
	Non‐oral	0	5	0	12	17
Total		37	17	25	16	95

**FIGURE 2:**
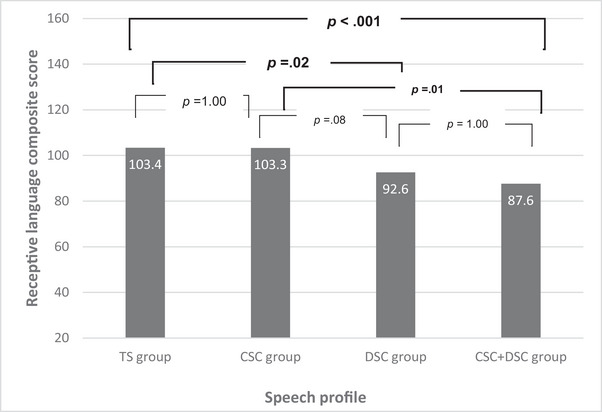
Receptive language scores illustrating group differences for those with typical speech (TS), cleft speech characteristics (CSC) only, developmental speech characteristics (DSC) only, and CSC + DSC.

**FIGURE 3:**
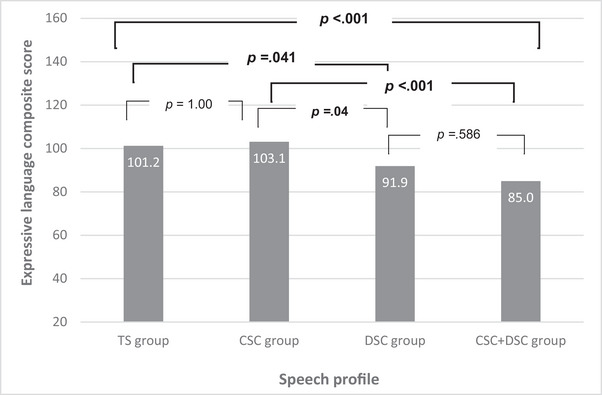
Expressive language scores illustrating group differences for those with typical speech (TS), cleft speech characteristics (CSC) only, developmental speech characteristics (DSC) only, and CSC + DSC.

Receptive and expressive language composite scores were analysed by speech profile. Group differences were explored through ANOVA analysis with post‐hoc Bonferroni corrections. For receptive language, there was a main effect of group (*F*(3, 91) = 7.52, *p* < 0.001, *η* ^2^ = 0.199) and statistically significant group differences were seen between participants with combined CSC + DSC and those with typically developing speech or CSC only. For expressive language, there was also a main effect of group (*F*(3, 91) = 8.39, *p* < 0.001, *η* ^2^ = 0.217) and statistically significant group differences were seen between participants with DSC (±CSC) and those with typically developing speech or CSC only. There was no difference in language scores for those with CSC only and typically developing speech and those with combined CSC + DSC consistently scored the lowest (Figures 2 and 3).

ANOVA analysis showed a main effect of group for all subtests with moderate to large effect sizes for all subtests (Table 6). After Bonferroni corrections, no significant differences across groups were seen for the Following Directions and Recalling Sentences subtests. However, statistically significant differences between participants with DSC (±CSC) and those with speech typical for their age or CSC only were found in all other subtests. Further details are presented in Appendix B.

**TABLE 6:** Descriptive statistics and analysis of variance (ANOVA) analysis for scaled scores from language subtests (from CELF‐5^UK^; Wiig et al., 2013) for those with typical speech (TS), cleft speech characteristics (CSC) only, developmental speech characteristics (DSC) only and CSC + DSC.

				ANOVA		
		*n*	Mean (SD, minimum‐maximum)	*F*(3, 91)	*p*	*η* ^2^ ^**^
Sentence comprehension	TS group	37	11.1 (2.6, 7−16)	9.64	< 0.001^*^	0.241
	CSC group	17	11.4 (3.4, 7−17)			
	DSC group	25	9.2 (3.2, 1−15)			
	CSC + DSC group	16	7.0 (2.1, 2−10)			
Word structure	TS group	37	11.9 (2.3, 8−17)	6.18	< 0.001^*^	0.169
	CSC group	17	12.1 (2.5, 8−17)			
	DSC group	25	10.4 (3.6, 3−18)			
	CSC + DSC group	16	8.4 (3.7, 1−14)			
Word classes	TS group	37	10.5 (2.9, 5−17)	4.02	0.010^*^	0.117
	CSC group	17	10.1 (2.9, 5−16)			
	DSC group	25	8.8 (2.4, 4−14)			
	CSC + DSC group	16	8.1 (1.9, 5−11)			
Following directions	TS group	37	9.9 (3.1, 4−17)	3.03	0.033^*^	0.091
	CSC group	17	10.1 (2.3, 7−14)			
	DSC group	25	8.0 (2.5, 4−15)			
	CSC + DSC group	16	8.8 (2.7, 5−13)			
Formulated sentences	TS group	37	9.0 (3.0, 3−15)	7.55	< 0.001^*^	0.199
	CSC group	17	8.9 (2.4, 5−14)			
	DSC group	25	7.1 (1.9, 2−10)			
	CSC + DSC group	16	5.6 (3.1, 1−11)			
Recalling sentences	TS group	37	9.6 (2.1, 6−16)	3.83	0.012^*^	0.112
	CSC group	17	9.9 (2.3, 7−15)			
	DSC group	25	8.2 (2.4, 4−14)			
	CSC + DSC group	16	8.1 (2.3, 6−13)			

*Note*: ^*^Statistically significant (*p* < 0.05); ^**^effect size: small effect = 0.01; medium effect = 0.06; large effect = 0.14.

### Post‐hoc analyses: Risk of low language scores across different SSD subtypes

A significant relationship between speech profiles and language scores was observed. This is an important clinical finding as it highlights a group within the CP ± L population who may be more at risk of language difficulties. Therefore, a post‐hoc binary logistic regression was conducted to ascertain the odds of having language skills below 1 SD for the differing speech profiles. Two binary language status variables were created coding low language/typical language with those scoring a standard score of 85 or below as low and those 86 or above as typical on both receptive and expressive language indices. Initially, speech profile was entered into the model as a predictor of language outcome. The model was run a second time adjusting for the following potential confounds: gender, socio‐economic status, and non‐verbal skills. In the first model, the presence of DSC (±CSC) was a significant predictor of language outcome, with those with phonological errors three to four times more likely to have receptive language scores below 1 SD from the mean compared with the group with typical speech, and those with a combined CSC + DSC over 13 times more likely to have expressive language scores below 1 SD from the mean compared with the group with typical speech. After adjusting for gender, SES and non‐verbal skills, the presence of combined CSC + DSC remained a significant predictor of expressive language outcome, with participants with CSC + DSC over 10 times more likely to have low expressive language scores compared with the group with typical speech for their age. However, confidence intervals for this group were wide, possibly due to small numbers, so results should be read with caution. Speech profile was not significantly associated with receptive language outcome, with non‐verbal skills more closely associated with receptive language. The results of the logistic regression for both receptive and expressive language outcome are shown in Table 7.

**TABLE 7:** Logistic regression exploring predictors of language outcomes measured using receptive and expressive composite language scores from the CELF‐5^UK^ (Wiig et al., 2013) for those with typical speech (TS), cleft speech characteristics (CSC) only, developmental speech characteristics (DSC) only and CSC + DSC.

		Model 1			Model 2		
		Odds ratio	95% CI	*p‐*value	Odds ratio	95% CI	*p*‐value
Receptive language^a^	TS	Reference	Reference	–	Reference	Reference	–
	CSC	0.32	0.04, 2.92	0.314	0.22	0.02, 2.20	0.196
	DSC	3.44	1.05, 11.27	0.041^*^	1.56	0.36, 6.69	0.553
	CSC + DSC	4.02	1.08, 15.03	0.039^*^	3.06	0.66, 14.13	0.151
	Gender	–	–	–	0.62	0.20, 1.96	0.414
	Socio‐economic status^c^	–	–	–	0.89	0.73, 1.08	0.228
	Non‐verbal skills^d^	–	–	–	0.38	0.22, 0.63	< 0.001^*^
Expressive language^b^	TS	Reference	Reference	–	Reference	Reference	–
	CSC	1.10	0.18, 6.68	0.918	0.88	0.14, 5.54	0.888
	DSC	3.21	0.83, 12.45	0.092	1.84	0.41, 8.19	0.423
	CSC + DSC	13.75	3.23, 58.59	< 0.001^*^	10.82	2.42, 48.32	0.002^*^
	Gender	–	–	–	0.92	0.31, 2.74	0.884
	Socio‐economic status^c^	–	–	–	0.89	0.74, 1.08	0.233
	Non‐verbal skills^d^	–	–	–	0.69	0.43, 1.11	0.129

*Note*: ^a^Naglekerke *R* ^2^ = 0.17 (model 1); *R* ^2^ = 0.42 (model 2).

^b^ Naglekerke *R* ^2^ = 0.24 (model 1); *R* ^2^ = 0.29 (model 2).

^c^ Index of Multiple Deprivation decile for England derived from postcode (Ministry of Housing, Communities and Local Government, 2019).

^d^ Raven's Coloured Progressive Matrices assessment (Raven, 1988).

^*^Statistically significant (*p* < 0.05).

## DISCUSSION

The CLAS study investigated the language and auditory skills of children aged 5–8 years with non‐syndromic CP ± L and their relationship to speech outcomes. This paper discusses the results from the language measures from the CLAS study.

The language profile of children with CP ± L as a group did not differ from normative samples (research question 1). On average, the language profile of the participants with CP ± L in this study showed skills to be within the average range for their age for receptive and expressive indices and all subtests of the CELF‐5^UK^.

Children with CP ± L and SSD did have poorer language skills than those with typically developing speech (research question 2). There was an association between speech production and language skills, with those presenting with an SSD scoring lower on all language tasks than those with speech typical for their age. There was however no direct relationship between language skills and the severity of the SSD as shown by the linear regression analyses.

A significant association was observed between language skills and type of speech profile, or nature of the SSD (research question 3). The nature of speech errors was varied, and speech errors were not confined to CSC. Over 40% of participants presented with phonological errors or DSC. This concurs with other studies which have reported high levels of DSC (Nachmani et al., 2022; Willadsen et al., 2019). It also reflects a more varied array of speech profile in children with CP ± L than much of the current research literature and outcome measurement practices suggest and demonstrates the existence of a range of SSD subtypes as observed in the wider population (Broomfield & Dodd, 2004; Wren et al., 2012).

Participants with significant CSC only, or articulation disorder, had similar language scores to those with typically developing speech. However, those with DSC, or phonological disorder, had poorer language skills. When adjusting for variables known to impact speech in children with CP ± L (e.g., cleft type and structurally related speech difficulties/history of speech surgery), no direct relationship between language skills and speech production was observed. However, participants presenting with a combined articulation and phonological speech disorder (CSC + DSC) were over ten times more likely to have expressive language difficulties. This is not surprising given the evidence from the non‐CP ± L literature. Studies investigating the underlying nature of SSD have shown an association between speech and language. Broomfield and Dodd (2004) reported lower levels of language difficulty in children presenting with articulation difficulties compared with those with phonological difficulties, and that these difficulties increased with the complexity of the nature of the SSD. In a study using cohort data on over 900 children at age 8 years, Wren et al. (2012) showed that where there was a relationship between the linguistically based task of non‐word repetition and a speech profile characterized by phonological processes such as substitutions and omissions, there was no such relationship with articulation difficulties. In contrast the severity of early SSD has not been found to be associated with later language or literacy skills. For example, Lewis et al. (2019), in a retrospective longitudinal study, found that a single‐word articulation assessment producing a PCC score in the pre‐school years was not predictive of long‐term outcomes in terms of persistent speech disorder or measures of language and literacy skills. Highlighting the different nature of speech profiles and their impact on other skills has not previously been reported in children with CP ± L and has clinical implications (see below).

### Complex language skills

Whilst scores for the group as whole in this study were within the average range, of note were the relatively low scores observed for the Formulated Sentences subtest of the CELF5^UK^. Across all participants with or without SSD, 40% scored below 1 SD on this subtest, with a mean scaled score of 7.9 where the normative mean was 10. Observations across the speech profiles showed that the mean for those with DSC (±CSC) was below 1 SD with those with a combined articulation and phonological disorder scoring a mean average scaled score of 5.6. This subtest assesses the ability to formulate complex sentences in a given context, combining semantic and pragmatic understanding and ability to produce correct grammatical sentences in order to convey an effective message. Oral language disorders have been shown to impair reading comprehension (Hulme & Snowling, 2011), ability to use complex morphosyntax (Rescorla, 2005), and to produce comprehensive written expository accounts (Scott & Windsor, 2000). We suggest that language difficulties in general and these skills in particular be monitored in those considered to be at risk. Further research with children with CP ± L is warranted to understand the specific levels of difficulty in this more complex task, the potential impact on educational attainment and its utility in identifying children in need of additional support.

### Explaining linguistic deficits in children with CP ± L

Early language delay in children with CP ± L is often explained through delayed babble due to later palatal competence compared with peers or through the high prevalence of conductive hearing loss (Baylis et al., 2020; Chapman et al., 2001). This simple explanation may be adequate to account for late talkers, and studies using composite language measures often suggest that children with CP ± L catch up on language skills (Lancaster et al., 2020). However, the high levels of phonological errors seen beyond the age of 5 years in this study and others, in addition to the increased risk of low language levels in older children (Lancaster et al., 2020), suggest a level of linguistic deficit beyond a delay.

Brain studies have shown neurostructural differences in those with CP ± L compared with peers (Sandor‐Bajusz et al., 2022) and studies of genes responsible for speech and language disorder have found chromosomal anomalies which overlap with those found in people with CP ± L (Lewis et al., 2006; Saleem et al., 2019). This may suggest that some children with CP ± L have a congenital neurological deficit. It may however also be explained by a neuroconstructivist approach to development (Karmiloff‐Smith, 1998). Neuroconstructivist theory places an emphasis on the plasticity of the neonatal brain. Whilst certain neurological structures may be best for the processing of, for example, sound versus movement, they may not always be the paths that are taken by the developing brain. Children may compensate for either faulty biological structures or for poor input from their environment to achieve a developmental goal. In this way their own development and experience influences the creation of domain‐specific modules within their brain. We would hypothesize that for children born with CP ± L this might mean using different pathways to compensate for difficulties hearing or difficulties practising speech sounds. For some, intervention such as timely palate repair or grommet insertion may suffice to ensure development continues along the expected trajectory. Whilst clinicians strive to provide timely care, sometimes intervention may not be sufficiently timely to prevent compensatory development which may result in atypical processing. There may also be other factors which make compensation more difficult (e.g., cognitive skills such as attention or non‐verbal skills). In this way the developmental trajectory is altered resulting in either a delay, or skills based on atypical processing mechanisms which may manifest as later perhaps subtle and/or persistent difficulties. Development is complex and even more so in a population with any congenital anomalies. Longitudinal observational and intervention studies may elucidate this theory.

### Clinical implications

These findings have clinical significance. If persistent phonological errors are present, then intervention must address this aspect of speech development and not just focus on articulation. Furthermore, persistent phonological errors should alert SLTs to the need for additional assessment of language. Without this information we may be missing underlying difficulties, even if mild, which may exacerbate difficulties with speech and academic progress.

The high ratio of the more severe non‐oral CSC combining with DSC should also be highlighted clinically. An underlying linguistic disorder may influence outcome in terms of the severity and persistence of SSD in children with CP ± L. Prioritizing treatment of a severe non‐oral CSC through articulation therapy is understandable and commonplace, but being aware of the influence of the concomitant phonological disorder and incorporating phonological intervention may result in better outcomes. Differential response to interventions based on varying linguistic phonological profiles in non‐CP ± L SSD strongly supports the suggestion that tailored interventions are necessary to promote change (Crosbie et al., 2005; Dodd & Bradford, 2000). Encouragingly, interest in this tailored approach with children with CP ± L is growing (Alighieri et al., 2020, 2023).

### Strengths and limitations

The major strengths of this study were its relatively large sample size and comprehensive measurement of language. The sample size also allowed contributing factors to be examined whilst controlling for known confounds and enabled a detailed phenotyping of the speech and language profiles of children with CP ± L.

For an observational study, there was a good level of participation, with 95 participants. However, once the sample was divided into subgroups for further analysis, some numbers were small and so statistical analysis underpowered. Whilst statistically significant group differences were seen across the speech profiles, the numbers in the CSC only and combined CSC + DSC groups were under 20. Larger studies, perhaps with purposive sampling would be useful to further evaluate the findings of this study. A study designed to recruit participants with these differing profiles in sufficient numbers to test the patterns found here would provide a fully powered study. Longitudinal designs would also allow us to understand the interactions over time of speech and language development. The cross‐sectional design of this study has allowed us to examine and understand empirical data which will inform future longitudinal studies into the underlying mechanisms for speech and language development in children with CP ± L.

This study measured a broad range of skills and discussed the potential impact of difficulties in these areas. It is one of only two studies (see Boyce et al., 2018) to use a comprehensive assessment such as the CELF 5^UK^ to break down language skills and compare to normative data. This choice led to the identification of difficulties with complex syntax in over 40% of this group of children with CP ± L which have not been apparent in previous research. Whilst the CELF‐5^UK^ provided a comprehensive assessment of language, it should also be noted that the normative data upon which it is based consisted of over 37% of the sample with parents who had degree level education. Although parental education was not measured in this study, the Index of Multiple Deprivation was used as a proxy for socio‐economic status and 48% were in the lowest three indices. No association was seen between socio‐economic status and language scores in this study; however, the mean standard scores should be interpreted with this in mind.

## CONCLUSIONS

SSD related to CP ± L has traditionally been described as an articulation disorder resulting from the structural anomaly. Early language delay has been attributed to the necessary delay in correcting these structures and to a high prevalence of conductive hearing loss. This study has shown that the nature of SSD in children with CP ± L follows a similar distribution to the wider population, with some who present with articulation disorder only, some with phonological disorder and some with a combined disorder. There was a high number of participants with phonological disorder, with or without CSC, and these participants were more likely to present with poorer language skills. These findings have implications for the assessment, treatment and monitoring of speech and language in children with CP ± L. There needs to be a paradigm shift to reconsider SSD in CP ± L in the wider context with knowledge of potential comorbidities and the impact of these on progress with speech and in school.

## CONFLICT OF INTEREST STATEMENT

The authors have no conflicts of interest to declare.

## Data Availability

Data are available upon request at https://doi.org/10.25405/data.ncl.24581901

## APPENDIX A ASSESSMENT BATTERY FROM THE CLEFT LANGUAGE AND AUDITORY SKILLS (CLAS) STUDY

**Table:**

Measure	Name of assessment	Reference
Hearing	SHOEBOX^®^ QuickTest (v.5.6.5) audiometry screen	https://www.shoebox.md/
Non‐verbal skills	Raven'sTM Coloured Progressive Matrices (1998 Edition)	Raven (1988)
Speech	Diagnostic Evaluation of Articulation and Phonology (DEAP)	Dodd et al. (2002)
Language	Clinical Evaluation of Language Fundamentals—5th UK Edition (CELF‐5 UK)	Wiig et al. (2013)
Phonological awareness	Clinical Evaluation of Language Fundamentals—4th UK Edition (CELF‐4 UK) subtest	Semel et al. (2006)
Temporal auditory processing	Random Gap Detection Test (RGDT)	Keith et al. (2001)
Processing speech in noise	Auditory Figure Ground Tests at 0 and 8 dB differences (AFG0 and AFG8+))	Keith et al. (2009)
Auditory attention	Test of Variables of Attention (TOVA v.9.0)	Greenberg et al. (2018)
Parent report of listening and processing skills	Evaluation of Children's Listening and Processing Skills (ECLiPS) questionnaire	Barry et al. (2015)

## APPENDIX B BONFERRONI POST‐HOC ANALYSIS FOR LANGUAGE SUBTESTS FOR THOSE WITH TYPICAL SPEECH, CLEFT SPEECH CHARACTERISTICS ONLY, DEVELOPMENTAL SPEECH CHARACTERISTICS ONLY AND CSC + DSC

**Table:**

Dependent variable			Mean difference	SE	Adjusted significance (*p*)	95% CI	
						Lower bound	Upper bound
Sentence comprehension	TS group	CSC group	−0.22	0.84	1.000	−2.48	2.05
		DSC group	1.90	0.74	0.074	−0.11	3.90
		DSC + CSC group	4.14^***^	0.86	**< 0.001**	1.82	6.45
	CSC group	TS group	0.22	0.84	1.000	−2.05	2.48
		DSC group	2.11	0.90	0.127	−0.32	4.54
		DSC + CSC group	4.35^***^	1.00	**< 0.001**	1.66	7.04
	DSC group	TS group	−1.90	0.74	0.074	−3.90	0.11
		CSC group	−2.11	0.90	0.127	−4.54	0.32
		DSC + CSC group	2.24	0.92	0.099	−0.23	4.71
	DSC + CSC group	TS group	−4.14^***^	0.86	**< 0.001**	−6.45	−1.82
		CSC group	−4.35^***^	1.00	**< 0.001**	−7.04	−1.66
		DSC group	−2.24	0.92	0.099	−4.71	0.23
Word structure	TS group	CSC group	−0.23	0.88	1.000	−2.59	2.14
		DSC group	1.53	0.77	0.305	−0.56	3.62
		DSC + CSC group	3.45^***^	0.89	**0.001**	1.04	5.87
	CSC group	TS group	0.23	0.88	1.000	−2.14	2.59
		DSC group	1.76	0.94	0.388	−0.78	4.29
		DSC + CSC group	3.68^**^	1.04	**0.004**	0.87	6.49
	DSC group	TS group	−1.53	0.77	0.305	−3.62	0.56
		CSC group	−1.76	0.94	0.388	−4.29	0.78
		DSC + CSC group	1.92	0.96	0.285	−0.66	4.50
	DSC + CSC group	TS group	−3.45^***^	0.89	**0.001**	−5.87	−1.04
		CSC group	−3.68^**^	1.04	**0.004**	−6.49	−0.87
		DSC group	−1.92	0.96	0.285	−4.50	0.66
Word classes	TS group	CSC group	0.43	0.77	1.000	−1.65	2.51
		DSC group	1.69	0.68	0.091	−0.15	3.52
		DSC + CSC group	2.36^*^	0.79	**0.021**	0.24	4.48
	CSC group	TS group	−0.43	0.77	1.000	−2.51	1.65
		DSC group	1.26	0.83	0.789	−0.97	3.49
		DSC + CSC group	1.93	0.92	0.226	−0.54	4.41
	DSC group	TS group	−1.69	0.68	0.091	−3.52	0.15
		CSC group	−1.26	0.83	0.789	−3.49	0.97
		DSC + CSC group	0.68	0.84	1.000	−1.60	2.95
	DSC + CSC group	TS group	−2.36^*^	0.79	**0.021**	−4.48	−0.24
		CSC group	−1.93	0.92	0.226	−4.41	0.54
		DSC group	−0.68	0.84	1.000	−2.95	1.60
Following directions	TS group	CSC group	−0.20	0.81	1.000	−2.37	1.98
		DSC group	1.88	0.71	0.059	−0.04	3.80
		DSC + CSC group	1.11	0.82	1.000	−1.12	3.33
	CSC group	TS group	0.20	0.81	1.000	−1.98	2.37
		DSC group	2.08	0.87	0.111	−0.26	4.41
		DSC + CSC group	1.31	0.96	1.000	−1.28	3.89
	DSC group	TS group	−1.88	0.71	0.059	−3.80	0.04
		CSC group	−2.08	0.87	0.111	−4.41	0.26
		DSC + CSC group	−0.77	0.88	1.000	−3.15	1.61
	DSC + CSC group	TS group	−1.11	0.82	1.000	−3.33	1.12
		CSC group	−1.31	0.96	1.000	−3.89	1.28
		DSC group	0.77	0.88	1.000	−1.61	3.15
Formulated sentences	TS group	CSC group	0.06	0.78	1.000	−2.05	2.17
		DSC group	1.88^*^	0.69	**0.047**	0.02	3.74
		DSC + CSC group	3.38^***^	0.80	**< 0.001**	1.22	5.53
	CSC group	TS group	−0.06	0.78	1.000	−2.17	2.05
		DSC group	1.82	0.84	0.195	−0.44	4.08
		DSC + CSC group	3.32	0.93	**0.003**	0.81	5.82
	DSC group	TS group	−1.88^*^	0.69	**0.047**	−3.74	−0.02
		CSC group	−1.82	0.84	0.195	−4.08	0.44
		DSC + CSC group	1.50	0.85	0.500	−0.81	3.80
	DSC + CSC group	TS group	−3.38^***^	0.80	**< 0.001**	−5.53	−1.22
		CSC group	−3.32^**^	0.93	**0.003**	−5.82	−0.81
		DSC group	−1.50	0.85	0.500	−3.80	0.81
Recalling sentences	TS group	CSC group	−0.23	0.66	1.000	−2.02	1.55
		DSC group	1.45	0.59	0.091	−0.13	3.03
		DSC + CSC group	1.59	0.68	0.127	−0.24	3.41
	CSC group	TS group	0.23	0.66	1.000	−1.55	2.02
		DSC group	1.68	0.71	0.120	−0.23	3.60
		DSC + CSC group	1.82	0.79	0.139	−0.30	3.94
	DSC group	TS group	−1.45	0.59	0.091	−3.03	0.13
		CSC group	−1.68	0.71	0.120	−3.60	0.23
		DSC + CSC group	0.14	0.72	1.000	−1.81	2.09
	DSC + CSC group	TS group	−1.59	0.68	0.127	−3.41	0.24
		CSC group	−1.82	0.79	0.139	−3.94	0.30
		DSC group	−0.14	0.72	1.000	−2.09	1.81

*Note*: TS, typical speech; CSC, cleft speech characteristics only; DSC, developmental speech characteristics only.

^*^ *p* < 0.05; ^**^ *p* < 0.01; ^***^ *p* ≤ 0.001.
